# Biomarker expression and survival in patients with non-small cell lung cancer receiving adjuvant chemotherapy in Denmark

**DOI:** 10.1371/journal.pone.0284037

**Published:** 2023-04-11

**Authors:** Tapashi Dalvi, Mette Nørgaard, Jon P. Fryzek, Naimisha Movva, Lars Pedersen, Hanh Pham Hansen, Jill Walker, Anita Midha, Norah Shire, Anne-Marie Boothman, James Rigas, Anders Mellemgaard, Torben R. Rasmussen, Stephen Hamilton-Dutoit, Deirdre Cronin-Fenton

**Affiliations:** 1 AstraZeneca, One MedImmune Way, Gaithersburg, Maryland, United States of America; 2 Department of Clinical Epidemiology, Aarhus University Hospital, Aarhus N, Denmark; 3 EpidStrategies, Rockville, Maryland, United States of America; 4 Institute of Pathology, Aarhus University Hospital, Aarhus N, Denmark; 5 AstraZeneca, Cambridge, United Kingdom; 6 Department of Oncology, Bornholm Hospital, Rønne, Denmark; 7 Dansk Lunge Cancer Gruppe, Odense, Denmark; 8 Department of Respiratory Medicine, Aarhus University Hospital, Aarhus N, Denmark; Abu Dhabi University, UNITED ARAB EMIRATES

## Abstract

**Introduction:**

Programmed cell death ligand-1 (PD-L1) expression may help identify patients with non-small cell lung cancer (NSCLC) who would benefit from immunotherapy. We assessed PD-L1 expression, and epidermal growth factor receptor (*EGFR*) and V-Ki-Ras2 Kirsten rat sarcoma (*KRAS*) mutations in NSCLC patients receiving adjuvant chemotherapy.

**Methods:**

Data for stage IB/II/IIIA NSCLC patients (diagnosed: 2001–2012) were retrieved from Danish population-based registries. Tumor tissue samples were tested for PD-L1 expression using VENTANA PD-L1 (SP263) Assay in tumor cells (TC) at ≥25% cutoff and immune cells (IC) at ≥1% and ≥25% cutoffs. *KRAS* and *EGFR* mutations were tested using PCR-based assays. Follow-up began 120 days after diagnosis until death/emigration/January 1, 2015, whichever came first. Using Cox proportional hazard regression, hazard ratios (HRs) were computed for overall survival (OS) for each biomarker, adjusting for age, sex, histology, comorbidities, and tissue specimen age.

**Results:**

Among 391 patients identified, 40.4% had stage IIIA disease, 49.9% stage II, and 8.7% stage IB. PD-L1-TC was observed in 38% of patients, *EGFR* mutations in 4%, and *KRAS* mutations in 29%. *KRAS* mutations were more frequent among patients with PD-L1 TC≥25% versus TC<25% (37% versus 24%). OS was not associated with PD-L1 TC≥25% versus TC<25% (stage II: adjusted HR = 1.15 [95% confidence interval: 0.66–2.01]; stage IIIA: 0.72 [0.44–1.19]). No significant association was observed with OS and PD-L1-IC ≥1% and ≥25%. *EGFR* and *KRAS* mutations were not associated with a prognostic impact.

**Conclusion:**

A prognostic impact for NSCLC patients receiving adjuvant chemotherapy was not associated with PD-L1 expression, or with *EGFR* and *KRAS* mutations.

## Introduction

Lung cancer is the leading cause of cancer-related mortality, with 1.8 million deaths estimated worldwide in 2018, representing 18.4% of total deaths from cancer [1]. The 5-year survival rates for lung cancer for the years 1999–2007 were 13.0% in Europe and 10.3% in Denmark [2]. Survival rates have improved over time, with more recent data (2012–2016) from Denmark indicating age-standardized 5-year relative survival rates for lung cancer of 14% in men and 20% in women [3].

Non-small cell lung cancer (NSCLC) accounts for approximately 85% of all lung cancer cases [4]. The European Society for Medical Oncology recommends surgery for the treatment of patients with early-stage, resectable NSCLC [5]. Patients with stage II/III NSCLC receiving adjuvant chemotherapy have an overall absolute survival improvement of 4%–5% at 5 years compared with those undergoing surgery alone [6]. Despite surgical resection with curative intent, approximately half of the patients with early-stage NSCLC experience disease relapse [6,7], and approximately 30%–55% of patients die from relapse [8]. Multiple immunotherapy agents targeting the programmed cell death-1 (PD-1)/programmed cell death ligand-1 (PD-L1) pathway have demonstrated clinical benefit and are approved for the treatment of patients with locally advanced/metastatic NSCLC [9,10]. For instance, pembrolizumab, a humanized anti-PD-1 antibody, in combination with pemetrexed and platinum chemotherapy demonstrated a survival benefit compared with pemetrexed and platinum chemotherapy plus placebo (median overall survival (OS): not reached versus 11.3 months) at a median follow-up of 10.5 months [11]. Similarly, durvalumab, an anti-PD-L1 antibody, prolonged median OS compared with placebo (not reached versus 28.7 months; median follow-up: 25.2 months) in patients with stage III, unresectable NSCLC and no disease progression after concurrent chemoradiotherapy [12].

PD-L1 expression on tumor cells (TCs) correlates with poor survival in melanoma, and urothelial, pancreatic, hepatocellular, and ovarian carcinomas [13–17]. In addition to being a prognostic marker, PD-L1 may also be useful for predicting patients who might benefit from anti-PD-1/PD-L1 therapies.

NSCLC tumors also harbor mutations in genes such as epidermal growth factor receptor (*EGFR*) and V-Ki-Ras2 Kirsten rat sarcoma (*KRAS*) [18,19]. Along with PD-L1 expression, such mutations can help further characterize patient populations. Mutant forms of *EGFR* upregulate PD-L1 expression in NSCLC, providing a direct link between oncogenic drivers and immune blockade [20]. Clinical data examining the association of PD-L1 expression and *EGFR* and *KRAS* biomarkers with survival in NSCLC may help elucidate the interplay between these biomarkers and any potential effect on patient prognosis.

Using a cohort of patients with stage IB/II/IIIA NSCLC receiving adjuvant chemotherapy, identified through population-based and medical registries in Denmark, we characterized PD-L1 expression in conjunction with EGFR and KRAS mutations, and their association with survival. Our primary objective was to assess PD-L1 expression in tumors by stage. Our secondary objectives were to evaluate OS by PD-L1 expression level on TCs, and by *KRAS* and *EGFR* mutation status, and by overlap between PD-L1 expression and *EGFR* and/or *KRAS* mutations. OS by PD-L1 expression on immune cells (ICs) and by percent of tumor-infiltration with ICs were evaluated as exploratory objectives. Understanding the prevalence and role of PD-L1, EGFR, and KRAS in adjuvant chemotherapy will provide a historical context for researchers, regulators, physicians, and payers as new trials are being designed and new drugs are being approved.

## Methods

### Study design and patients

We ascertained data on patients, formalin-fixed, paraffin-embedded (FFPE) tumor tissue availability, clinical characteristics, and vital status through the following Danish registries linked on an individual-level via the civil personal registration (CPR) number: the Danish Cancer Registry of mandatory cancer reporting [21,22]; the Danish Lung Cancer Registry of all lung cancers diagnosed in Denmark since 2000 [23]; the Danish National Patient Registry [24,25], which contains inpatient, outpatient, and emergency room visit information; the Danish National Pathology Registry, which contains detailed nationwide records of all pathology specimens analyzed in Denmark [26]; and the Civil Registration System, an administrative register that includes sex and dates of birth and death [27] (S1 Fig). The study was granted ethical approval by the Scientific Ethical Committee of the Central Denmark Region (reference number: 1-10-72-14-15). The use of registry-based data in this study was based on the General Data Protection Regulation Art. 9 (2), j), Art. 6 (1), e); and the Danish Data Protection Law §11. Due to the use of archival tissue and registry-based data, informed consent was not required. We had access to CPR numbers for data linkage purposes. No other personally identifiable information was used.

We included Danish citizens (aged ≥18 years) with NSCLC diagnosed between 2001 and 2012 and FFPE primary tumor tissue available from pathology archives [26] for the analysis of molecular markers. Receipt of adjuvant chemotherapy was defined as surgery within the first 60 days of diagnosis of stage IB/II/IIIA NSCLC and initiation of chemotherapy within 120 days of diagnosis. No other clinical or biological criteria were used for patient selection. We selected eligible patients from 2012 backwards until the target number of patients were enrolled.

### Tumor samples and biomarker assessment

Using the Danish National Pathology Registry, we identified FFPE tumor tissue samples from study patients, comprising diagnostic biopsies or surgical resection specimens. Representative tissue blocks were obtained from the pathology archives of hospitals throughout Denmark. FFPE blocks, which are routinely used for immunohistochemistry (IHC) analyses, were sectioned at a thickness of 4–5 μm and mounted on positively charged slides. The study pathologist reviewed a hematoxylin and eosin–stained section to confirm the original NSCLC diagnosis and to ensure that specimens contained ≥100 TCs per section. Labeled slides were shipped at ambient temperature to Hematogenix Laboratory Services (Tinley Park, IL) for the staining and scoring of PD-L1 expression levels using the VENTANA PD-L1 (SP263) Assay (Ventana Medical Systems Pvt. Ltd., Tucson, AZ) according to the manufacturer’s instructions and a predefined scoring algorithm by manufacturer-trained pathologists [28,29]. We evaluated PD-L1 expression levels on the TC membrane and on tumor-associated ICs at cutoffs of 25% and 1% (TC ≥25%, IC ≥25%, and IC ≥1%) based on the clinical trial protocols [30] at that time and the VENTANA PD-L1 assay limitations. Tumor infiltration by immune cells, irrespective of PD-L1 expression, was provided as a percent of total tumor-associated ICs. We detected mutations in the *KRAS* (exons 2 and 3: codon 12, codon 13, or codon 61 mutations) and *EGFR* (exons 18–21: G719X, S768I, T790M, L858R, exon 19 deletion, or exon 20 insertion) genes in tumor tissues using the Cobas^®^ polymerase chain reaction (PCR)-based system (Roche Molecular Systems Inc, Branchburg, NJ, USA; CE-IVD 5852170190, CE-IVD 6471463190). We extracted and purified tumor DNA using the Cobas® DNA Sample Preparation Kit (Roche Molecular Systems Inc., Branchburg, NJ) for FFPE tumor tissue specimens according to the manufacturer’s instructions.

### Study variables and covariates

Registry covariates of interest included tissue specimen age, patient age at diagnosis (as a continuous variable), sex, American Joint Committee on Cancer stage at diagnosis (7^th^ edition), tumor site, histology (adenocarcinoma or other [i.e. squamous, large-cell, adenosquamous, and non-small carcinomas, and carcinoids]), Eastern Cooperative Oncology Group performance status (ECOG PS), comorbidities (recorded up to 10 years before NSCLC diagnosis and registered in the Danish National Patient Registry) using Charlson Comorbidity Index, and smoking status.

### Statistical analysis

We provided descriptive statistics for all covariates of interest and constructed Kaplan-Meier curves for each survival outcome stratified by PD-L1 expression level category and *EGFR* and *KRAS* mutation status. OS was defined as the time from 120 days after primary diagnosis to death due to any cause. We used Cox proportional hazard regression analysis to compute hazard ratios (HRs) and associated 95% confidence intervals (CIs) adjusting for age, sex, comorbidities, tissue specimen age, and adenocarcinoma histology (versus other). The Breslow and Efron methods for Cox regression were used for handling ties. Follow-up began 120 days after diagnosis and continued until death, emigration, or the end of follow-up (January 1, 2015), whichever came first. Patients without an event during follow-up were censored at the end of the follow-up period. For patients with stage IB NSCLC, the numbers were too small to conduct multivariable analyses and not described further.

## Results

**Fig 1:**
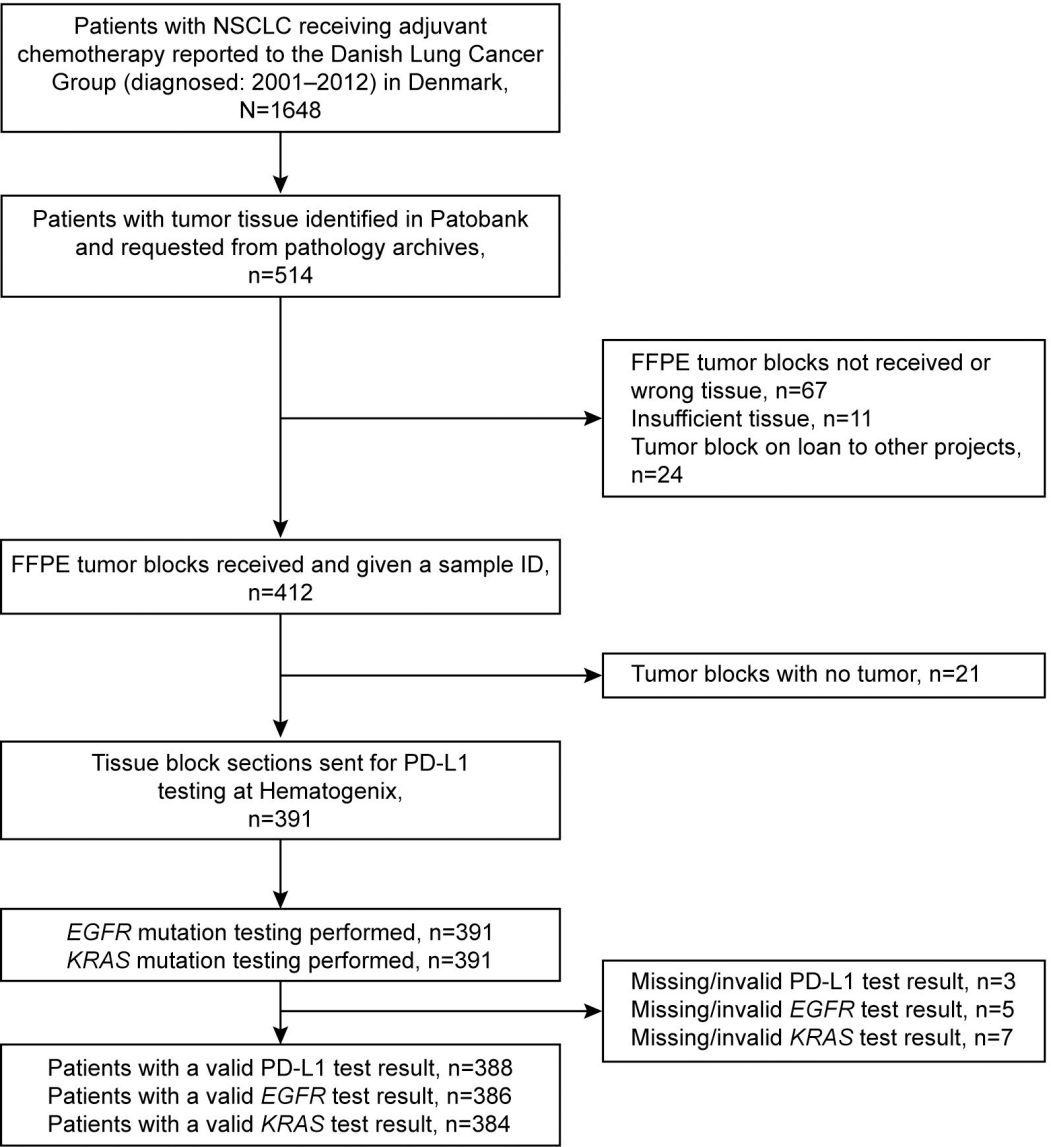
Patient disposition. EGFR, epidermal growth factor receptor; FFPE, formalin-fixed, paraffin-embedded; KRAS, V-Ki-Ras2 Kirsten rat sarcoma; NSCLC, non-small cell lung cancer; PD-L1, programmed cell death ligand-1.

Our study cohort included 391 patients with NSCLC who received adjuvant chemotherapy (Fig 1). Overall, 40% of patients were diagnosed with stage IIIA disease, 50% with stage II, and 9% with stage IB.

### PD-L1 expression

PD-L1 TC ≥25% and TC <25% was observed in 38% and 62% of patients, respectively (Table 1). Patients with PD-L1 TC ≥25% compared with those with PD-L1 TC <25% were more likely to be <65 years old (62.0% versus 52%), female (55% versus 49%), and smokers (59% versus 47%). ECOG PS was better for patients with PD-L1 TC ≥25% compared with those with TC <25% (41% versus 28% were fully active without restrictions [ECOG PS grade 0]). Adenocarcinoma was the most common histological subtype across all patients, irrespective of their PD-L1 expression levels (approximately 60%).

**Table 1:** Baseline demographics, disease characteristics, and medical history of patients with NSCLC receiving adjuvant chemotherapy by PD-L1 expression level, *EGFR* mutation, and *KRAS* mutation.

Characteristic	Overall N = 391	PD-L1 TC ≥25% n = 147	PD-L1 TC <25% n = 241	*EGFR* mutation n = 14	*EGFR* wild-type n = 372	*KRAS* mutation n = 112	*KRAS* wild-type n = 272
**Age, median (range), years**	64.0 (40.0–80.0)	62.0 (43.0–80.0)	64.0 (40.0–78.0)	62.0 (48.0–78.0)	64.0 (40.0–80.0)	63.0 (43.0–79.0)	64.0 (40.0–80.0)
**Age group, n (%), years**							
**18–59**	141 (36.1)	59 (40.1)	82 (34.0)	5 (35.7)	134 (36.0)	49 (43.8)	87 (32.0)
**60–64**	75 (19.2)	32 (21.8)	43 (17.8)	<5^a^	70 (18.8)	15 (13.4)	60 (22.1)
**65–69**	96 (24.6)	29 (19.7)	66 (27.4)	<5^a^	91 (24.5)	28 (25.0)	66 (24.3)
**≥70**	79 (20.2)	27 (18.4)	50 (20.7)	<5^a^	77 (20.7)	20 (17.9)	59 (21.7)
**Male, n (%)**	191 (48.8)	66 (44.9)	123 (51.0)	5 (35.7)	184 (49.5)	37 (33.0)	151 (55.5)
**Smoking history, n (%)**							
**Non-smoker**	8 (2.0)	<5^a^	7 (2.9)	0 (0.0)	8 (2.2)	<5^a^	7 (2.6)
**Smoker**	200 (51.2)	86 (58.5)	114 (47.3)	7 (50.0)	190 (51.1)	61 (54.5)	136 (50.0)
**Missing**	183 (46.8)	-^a^	120 (49.8)	7 (50.0)	174 (46.8)	-^a^	129 (47.4)
Nicotine use, median (min–max), pack-years	36 (0–125)	35 (0–100)	40 (0–125)	30 (18–50)	36 (0–125)	35 (0–66)	40 (0–125)
**ECOG PS, n (%)**							
**0**	127 (32.5)	60 (40.8)	67 (27.8)	<5^a^	119 (32.0)	40 (35.7)	85 (31.3)
**1**	64 (16.4)	25 (17.0)	39 (16.2)	<5^a^	63 (16.9)	15 (31.4)	49 (18.0)
**2/3**	20 (5.1)	<5^a^	18 (7.5)	<5^a^	19 (5.1)	6 (5.4)	12 (4.4)
**Missing/unknown**	180 (46.0)	-^a^	117 (48.5)	8 (57.1)	171 (46.0)	51 (45.5)	126 (46.3)
**Stage at start of follow-up, n (%)**							
**I**	34 (8.7)	13 (8.8)	21 (8.7)	<5^a^	30 (8.1)	NR^b^	20 (7.4)
**II**	195 (49.9)	74 (50.3)	119 (49.4)	6 (42.9)	187 (50.3)	56 (50.0)	136 (50.0)
**III**	158 (40.4)	NR^c^	98 (40.7)	5(35.7)	151 (40.6)	NR^b^	114 (41.9)
**Missing**	<5^a^	<5^a^	<5^a^	-^a^	<5^a^	<5^a^	<5^a^
**Histology type, n (%)**							
**Squamous**	123 (31.5)	47 (32.0)	76 (31.5)	<5^a^	121 (32.5)	5 (4.5)	116 (42.6)
**Adenocarcinoma**	239 (61.1)	88 (59.9)	148 (61.4)	11 (78.6)	225 (60.5)	98 (87.5)	137 (50.4)
**Other** ^**d**^	29 (7.4)	12 (8.2)	17 (7.1)	<5^a^	26 (7.0)	9 (8.0)	19 (7.0)
**Primary tumor location, n (%)**							
**Right lung**	108 (27.6)	45 (30.6)	63 (26.1)	<5^a^	104 (28.0)	36 (32.1)	71 (26.1)
**Left lung**	115 (29.4)	44 (29.9)	71 (29.5)	6 (42.9)	108 (29.0)	28 (25.0)	84 (30.9)
**Missing**	168 (43.0)	58 (39.5)	107 (44.4)	-^a^	160 (43.0)	48 (42.9)	117 (43.0)
Charlson comorbidity at diagnosis,^e^ n (%)							
**Myocardial infarction**	23 (5.9)	<5^a^	19 (7.9)	0 (0.0)	23 (6.2)	<5^a^	19 (7.0)
**Congestive heart failure**	8 (2.0)	0 (0.0)	8 (3.3)	<5^a^	7 (1.9)	<5^a^	6 (2.2)
**Peripheral vascular disease**	23 (5.9)	6 (4.1)	17 (7.1)	0 (0.0)	23 (6.2)	8 (7.1)	15 (5.5)
**Cerebrovascular disease**	20 (5.1)	6 (4.1)	14 (5.8)	0 (0.0)	19 (5.1)	5 (4.5)	14 (5.1)
**Chronic pulmonary disease**	43 (11.0)	19 (12.9)	24 (10.0)	<5^a^	40 (10.8)	9 (8.0)	32 (11.8)
**Connective tissue disease**	13 (3.3)	<5^a^	10 (4.1)	<5^a^	11 (3.0)	<5^a^	8 (2.9)
**Ulcer disease**	25 (6.4)	9 (6.1)	15 (6.2)	<5^a^	23 (6.2)	<5	22 (8.1)
**Mild liver disease**	5 (1.3)	0 (0.0)	5 (2.1)	<5^a^	<5^a^	<5^a^	<5^a^
**Diabetes mellitus**	20 (5.1)	<5^a^	16 (6.6)	<5^a^	19 (5.1)	<5^a^	16 (5.9)
**Moderate/severe renal disease**	5 (1.3)	<5^a^	<5^a^	0 (0.0)	-^a^	<5^a^	<5^a^

^a^Some cells have less than 5 observations. In some cases, other cell-contents are not presented because one could easily back-calculate the sensitive data from that information.

^b^The numbers are too small to report, but a majority of these patients had *KRAS* mutations.

^c^The numbers are too small to report, but a majority of these patients had PD-L1 TC ≥25%.

^d^“Other” includes large-cell, adenosquamous, and non-small cell carcinomas and carcinoids.

^e^Patients categorized by the Charlson Comorbidity Index were not mutually exclusive.

ECOG PS, Eastern Cooperative Oncology Group performance status; *EGFR*, epidermal growth factor receptor; *KRAS*, V-Ki-Ras2 Kirsten rat sarcoma; max, maximum; min, minimum; NR, not reported; NSCLC, non-small cell lung cancer; PD-L1, programmed cell death ligand-1; TC, tumor cell.

### Overall Survival by PD-L1 expression level

**Fig 2:**
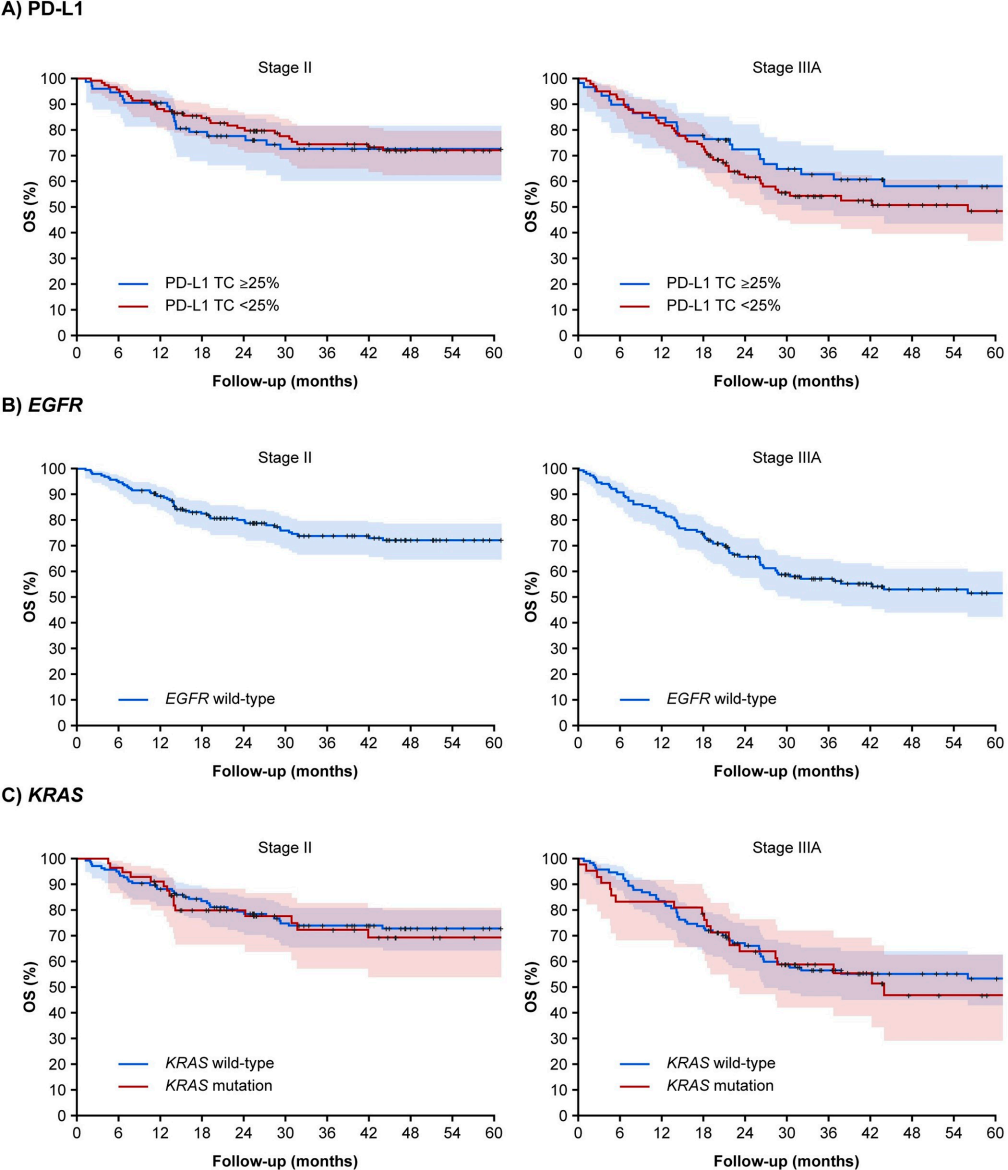
Kaplan-Meier curves for OS in patients with stage II and stage IIIA NSCLC receiving adjuvant chemotherapy according to PD-L1 expression (A), EGFR mutation status (B) and *KRAS* mutation status (C). ^a^Neither the survival estimate nor the upper confidence limit reached the 50^th^ percentile. CI, confidence interval; *EGFR*, epidermal growth factor receptor; *KRAS*, V-Ki-Ras2 Kirsten rat sarcoma; NR, not reached; NSCLC, non-small cell lung cancer; OS, overall survival; PD-L1, programmed cell death ligand-1; TC, tumor cell.

Median OS was not yet reached at the end of follow-up for patients with stage II NSCLC (Fig 2A). Among patients with stage IIIA NSCLC, median OS was 56 months (95% CI: 26.1–not reached) for patients with PD-L1 TC <25% and was not reached for those with PD-L1 TC ≥25%.

Adjusted survival analyses for PD-L1 TC ≥25% compared with PD-L1 TC <25% yielded an HR of 1.15 (95% CI: 0.66–2.01) in stage II and 0.72 (95% CI: 0.44–1.19) in stage IIIA. Analyses of PD-L1 IC expression levels at IC ≥25% yielded adjusted HRs of 0.78 (95% CI: 0.39–1.56) and 0.76 (95% CI: 0.43–1.34) in stages II and IIIA, respectively. PD-L1 expression levels at IC ≥1% yielded similar HRs in stages II and IIIA (0.72 [95% CI: 0.35–1.48] and 0.68 [95% CI: 0.38–1.22], respectively; Table 2). The percent of tumor infiltration by ICs was associated with an adjusted HR of 0.96 (95% CI: 0.92–1.00) in stage II and 0.97 (95% CI: 0.94–1.00) in stage IIIA (Table 2). Median survival was not reached, but the Kaplan-Meier estimate of 25% survival (i.e., the timepoint where 25% of the study population survived) was greater in patients with stage II NSCLC with PD-L1 TC<25% compared with those with PD-L1 TC ≥25%; however, among patients with stage IIIA NSCLC, those with PD-L1 TC ≥25% demonstrated longer survival (S1 Table).

**Table 2:** Adjusted HR for OS by biomarker status in patients with stage II or stage IIIA NSCLC receiving adjuvant chemotherapy.

	HR (95% CI)	
	Stage II	Stage IIIA
**PD-L1 TC ≥25% (ref: TC <25%)**	1.15 (0.66–2.01)	0.72 (0.44–1.19)
**PD-L1 IC ≥25% (ref: IC <25%)**	0.78 (0.39–1.56)	0.76 (0.43–1.34)
**PD-L1 IC ≥1% (ref: IC <1%)**	0.72 (0.35–1.48)	0.68 (0.38–1.22)
**% Tumor infiltration with ICs (continuous)**	0.96 (0.92–1.00)	0.97 (0.94–1.00)
*EGFR* mutation (ref: wild-type)	0.91 (0.22–3.79)	0.58 (0.08–4.30)
*KRAS* mutation (ref: wild-type)	1.54 (0.81–2.93)	0.88 (0.50–1.53)

Cox proportional hazard models were used to adjust for age (continuous), sex, adenocarcinoma histology (versus other), comorbidities, and tissue specimen age.

CI, confidence interval; *EGFR*, epidermal growth factor receptor; HR, hazard ratio; IC, immune cell; *KRAS*, V-Ki-Ras2 Kirsten rat sarcoma; NSCLC, non-small cell lung cancer; OS, overall survival; PD-L1, programmed cell death ligand-1; ref, reference; TC, tumor cell.

### EGFR mutation status

Few patients (3.6%) had a tumor with an *EGFR* mutation (Table 1). Patients with *EGFR* wild-type tumors compared with those with *EGFR*-mutated tumors were more likely to be ≥65 years old (45% versus 36%) and less likely to be female (51% versus 64%). Tumors with *EGFR* mutations were more likely to be associated with an adenocarcinoma histology compared with *EGFR* wild-type tumors (79% versus 61%).

### KRAS mutation status

Twenty-nine percent of patients had tumors with ≥1 *KRAS* mutation (Table 1). Patients with *KRAS*-mutated tumors were more likely to be female (67% versus 45%) and smokers (55% versus 50%) compared with patients without the mutation. Adenocarcinoma was the most common histology among patients with a *KRAS* mutation (88%). For *KRAS* wild-type tumors, adenocarcinoma (50%) and squamous (43%) histology were equally likely.

### Overall Survival by EGFR and KRAS mutations

Median OS among stage II patients with *EGFR* wild-type and mutated tumors and in stage IIIA patients with *EGFR* mutated tumors was not reached (Fig 2B). Adjusted survival analyses for *EGFR*-mutated tumors compared with wild-type tumors yielded an HR of 0.91 (95% CI: 0.22–3.79) in stage II and 0.58 (95% CI: 0.08–4.30) in stage IIIA (Table 2). Given the low frequency of patients with *EGFR* mutations, we were unable to draw conclusions regarding the association of *EGFR* mutations with survival. Median OS among patients with *KRAS*-mutated and wild-type tumors was not reached in stage II; however, for stage IIIA, median OS was 44 months (95% CI: 21.9–not reached) and 113.2 months (95% CI: 26.8–not reached), respectively (Fig 2C). Adjusted survival analyses for patients with *KRAS*-mutated tumors compared with wild-type tumors yielded an HR of 1.54 (95% CI: 0.81–2.93) and 0.88 (95% CI: 0.50–1.53) in stages II and IIIA, respectively (Table 2).

### Association of PD-L1 expression levels with EGFR and KRAS mutations

No associations were observed between PD-L1 expression in TCs at 25% cutoff and *EGFR* mutations (PD-L1 TC ≥25% versus PD-L1 TC <25%, <3% versus 5%, respectively). Patients with PD-L1 TC ≥25% were more likely to have tumors with a *KRAS* mutation than those with PD-L1 TC <25% (37% versus 24%; Table 3). Similar results were seen when patients were grouped by stage for *KRAS*-mutated tumors (38% versus 24% for stage II tumors); the numbers were too small to report for *EGFR*-mutated tumors by stage (S2 Table).

**Table 3:** PD-L1 expression level by *EGFR* and *KRAS* mutation status in patients with NSCLC receiving adjuvant chemotherapy^a^.

Characteristic	Overall N = 391	PD-L1 TC ≥25% n = 147	PD-L1 TC <25% n = 241
EGFR status, n (%)			
Mutation	14 (3.6)	<5^b^	-^b^
Wild-type	372 (95.1)	142 (96.6)	227 (94.2)
KRAS status, n (%)			
Mutation	112 (28.6)	54 (36.7)	58 (24.1)
Wild-type	272 (69.6)	91 (61.9)	178 (73.9)

^a^Individual numbers do not total to overall patients because of invalid/missing results.

^b^Some cells have less than 5 observations. In some cases, other cell-contents are not presented because one could easily back-calculate the sensitive data from that information.

*EGFR*, epidermal growth factor receptor; *KRAS*, V-Ki-Ras2 Kirsten rat sarcoma; NSCLC, non-small cell lung cancer; PD-L1, programmed cell death ligand-1; TC, tumor cell.

## Discussion

In tumors from patients with NSCLC who received adjuvant chemotherapy, a prognostic impact was not observed with PD-L1 expression at TC ≥25%/IC ≥25%/IC ≥1% cutoffs, or with *KRAS* mutations. However, tumors from patients who had PD-L1 TC ≥25%, were more likely to harbor *KRAS* mutations compared with those with PD-L1 TC<25%. Additionally, we observed no OS benefit with increasing tumor membrane infiltration with immune cells. Due to the low prevalence of patients with *EGFR* mutations, we could not evaluate their association with survival.

The NSCLC stage distribution in our study population was similar to that in the LuCaBIS (Lung Cancer Burden of Illness Study) population [31]. Most study patients smoked tobacco, as is observed in the general Danish population [32]. Notably, a history of smoking was more likely to be associated with PD-L1 TC ≥25% expression compared with PD-L1 TC <25%. However, no association between smoking and PD-L1 expression has been reported in patients with surgically resected early-stage or advanced NSCLC [33–35]. Previous reports indicate no consensus regarding a possible association between PD-L1 expression levels and tumor histology. One study showed PD-L1 expression to be associated with adenocarcinomas [36], while a second study reported an association with squamous carcinomas [37]. In contrast, consistent with our findings, two other studies of 204 [38] and 109 [39] patients each, with advanced NSCLC found no association between PD-L1 expression and tumor histology.

While we found PD-L1 expression at TC ≥25% in approximately one-third of study tumors, comparison with findings from other studies was challenging due to varying PD-L1 expression thresholds. In an observational study including 2634 patients with locally advanced/metastatic NSCLC from 18 countries, the percentage of tumors with PD-L1 expression at TC ≥50% was approximately 22% [40]. In a pooled analysis of tumor tissue samples from three KEYNOTE clinical trials assessing pembrolizumab efficacy and safety in patients with advanced NSCLC, PD-L1 expression at TC ≥50% was found in 28% of tumors [41]. However, in a study in Australia in patients with surgically resected early stage NSCLC (stages I–III), high PD-L1 expression (defined as tumors with ≥50% cells showing positive membrane staining with a monoclonal anti-PD-L1 antibody) was observed in only 7.4% of tumors; though PD-L1 staining in ≥1% of TCs and ≥5% of TCs was identified in 28% and 20% of cases [42]. Taken together, despite varying cutoffs, these reports suggest variability in PD-L1 expression, which may be related to the techniques used to measure PD-L1 levels or inherent variability in PD-L1 expression among different NSCLC cohorts.

We found no association between PD-L1 expression on TCs or ICs and OS across stages. This is in contrast to findings in an Australian study of patients with surgically resected early-stage NSCLC (n = 681), that high PD-L1 tumor expression (TC ≥50%) was associated with longer OS compared with low PD-L1 tumor expression (113.2 months versus 85.5 months) [42]. Of note, only a subset of patients in the latter study was expected to have received adjuvant chemotherapy. Conflicting findings have also been observed in advanced NSCLC. In a recent review of patients with advanced/metastatic NSCLC, PD-L1-TC expression was found to be associated with shorter OS in most of the studies evaluated [33]. Conversely, a study in advanced NSCLC populations from Denmark observed no association between PD-L1 expression (TC ≥50%) and OS [38]. The discrepancy in the survival outcomes with PD-L1 expression may be because of the differences in the patient populations and assay antibodies used in the studies. While analytical comparisons have demonstrated a reasonable agreement among most of the available PD-L1 detection assays (VENTANA PD-L1 [SP263] Assay, Dako PD-L1 IHC 22C3 pharmDx, and Dako PD-L1 IHC 28–8 pharmDx) [28], lower sensitivity has been reported with the VENTANA PD-L1 (SP142) Assay for determining tumor proportion scores on TCs in comparison with other assays [43]. However, none of the published studies have used the VENTANA PD-L1 (SP263) Assay, and in the current study, this assay had a very low failure rate (<1%), potentially because of the stringent requirements for adequate TC content in the tumor tissue samples tested.

Unlike in the current study, a few reports [36,44], including one in an NSCLC patient population specifically selected for *EGFR* mutations [44], have found an association between *EGFR* mutations and PD-L1 expression. As the prevalence of *EGFR* mutations in the current study population was low (3.6%), the study did not detect any potential associations between PD-L1 and *EGFR*. While we found that 29% of tumors had *KRAS* mutations, in other studies of surgically resected early-stage NSCLC, *KRAS* mutations have been reported at a lower frequency of 17%–20% [45]. A pooled analysis of studies among patients with resected early-stage NSCLC treated with adjuvant chemotherapy identified *KRAS* mutations in 19% of patient specimens [46]. We extended these findings by also evaluating the association of *KRAS* mutations with PD-L1 expression and found that *KRAS* mutations were more likely to be present in tumors with PD-L1 TC ≥25%.

Earlier reports indicate that the presence of tumor infiltration with lymphocytes favors a positive prognosis and improved survival in cancer, including patients with NSCLC in the adjuvant chemotherapy setting [47,48]. In the current exploratory analyses, increasing tumor infiltration with ICs was associated with a modest per-unit prognostic impact among patients requiring adjuvant chemotherapy for stage II and IIIA disease.

Our study had some limitations. Owing to the small number of patients with *EGFR* or *KRAS* mutations or those expressing PD-L1 above the cutoffs, careful interpretation of the biomarker status and related outcomes is warranted. Furthermore, there were too few patients with stage IB NSCLC receiving adjuvant therapy to draw any firm conclusions. Detailed information on adjuvant chemotherapy regimens was not available from the registry. PD-L1 TC was scored at a 25% TC cutoff only, therefore limiting comparison with other studies. The survival analyses were not adjusted for ECOG PS and smoking status because of a large proportion of missing data. Additionally, during the follow-up period, neither checkpoint inhibitors nor *EGFR* tyrosine kinase inhibitors were approved as adjuvant therapy. Only first-generation *EGFR*-targeting therapies were approved for the treatment of metastatic disease at the time of the study.

This study exhibits several strengths. Danish health registries are some of the world’s oldest registry systems and are used extensively for research [49]. Advantages of using these registries include a nationalized, free healthcare system with uniform disease and procedure registration and coding, and the ability to link and collect data on a given patient from multiple registries, resulting in a complete and unbiased follow-up for all cohort members [49].

Findings from this observational study suggest that OS of patients with NSCLC who received adjuvant chemotherapy is not associated with PD-L1 expression in TCs or ICs. *KRAS* mutations were more likely to be present in tumors with PD-L1 TC ≥25% compared with those with PD-L1 TC <25%, however, there was no prognostic impact observed with the presence of *KRAS* or *EGFR* mutations.

## Supporting information

S1 Fig Network of population-based medical registries linkable on an individual level via the CPR number. (DOCX)

S1 Table Kaplan-Meier estimates of 25% survival (months, 95% CI) by biomarker status in patients with stage II or stage IIIA NSCLC receiving adjuvant chemotherapy. (DOCX)

S2 Table PD-L1 expression level by *EGFR* and *KRAS* mutation status in patients with stages II, and IIIA NSCLC receiving adjuvant chemotherapy. (DOCX)

## References

[1] Bray F, Ferlay J, Soerjomataram I, et al. Global cancer statistics 2018: GLOBOCAN estimates of incidence and mortality worldwide for 36 cancers in 185 countries. CA Cancer J Clin. 2018;68: 394–424. doi: 10.3322/caac.21492 30207593

[2] De Angelis R, Sant M, Coleman MP, et al; EUROCARE-5 Working Group. Cancer survival in Europe 1999–2007 by country and age: results of EUROCARE—5-a population-based study. Lancet Oncol. 2014;15: 23–34. doi: 10.1016/S1470-2045(13)70546-1 24314615

[3] Danckert B, Ferlay J, Engholm G, et al. NORDCAN: Cancer Incidence, Mortality, Prevalence and Survival in the Nordic Countries, Version 8.2 (26.03.2019). Association of the Nordic Cancer Registries. Danish Cancer Society. https://www-dep.iarc.fr/NORDCAN/english/frame.asp. Accessed 15 June 2020.

[4] Herbst RS, Morgensztern D, Boshoff C. The biology and management of non-small cell lung cancer. Nature. 2018;553: 446–454. doi: 10.1038/nature25183 29364287

[5] Postmus PE, Kerr KM, Oudkerk M, et al; ESMO Guidelines Committee. Early and locally advanced non-small-cell lung cancer (NSCLC): ESMO Clinical Practice Guidelines for diagnosis, treatment and follow-up. Ann Oncol. 2017;28(suppl 4): iv1–iv21. doi: 10.1093/annonc/mdx222 28881918

[6] Rud AK, Borgen E, Mælandsmo GM, et al. Clinical significance of disseminated tumour cells in non-small cell lung cancer. Br J Cancer. 2013;109: 1264–1270. doi: 10.1038/bjc.2013.450 23942067 PMC3778301

[7] Pisters KM, Le Chevalier T. Adjuvant chemotherapy in completely resected non-small-cell lung cancer. *J Clin Oncol*. 2005;23: 3270–3278. Erratum in: J Clin Oncol. 2008;26: 2238. doi: 10.1200/JCO.2005.11.478 15886314

[8] Uramoto H, Tanaka F. Recurrence after surgery in patients with NSCLC. Transl Lung Cancer Res. 2014;3: 242–249. doi: 10.3978/j.issn.2218-6751.2013.12.05 25806307 PMC4367696

[9] Mielgo-Rubio X., et al. Immunotherapy in non-small cell lung cancer: Update and new insights. J Clin Transl Res. 2021 Jan 20;7(1): 1–21. 10.18053/jctres.07.202101.001. 34104805 PMC8177026

[10] Rocco D., et al. Pharmacodynamics of current and emerging PD-1 and PD-L1 inhibitors for the treatment of non-small cell lung cancer. Review Expert Opin Drug Metab Toxicol. 2020 Feb;16(2): 87–96. doi: 10.1080/17425255.2020.1721460 31978315

[11] Gandhi L, Rodríguez-Abreu D, Gadgeel S, et al. Pembrolizumab plus chemotherapy in metastatic non–small-cell lung cancer. N Engl J Med. 2018;378: 2078–2092. doi: 10.1056/NEJMoa1801005 29658856

[12] Antonia SJ, Villegas A, Daniel D, et al. Overall survival with durvalumab after chemoradiotherapy in stage III NSCLC. N Engl J Med. 2018;379: 2342–2350. doi: 10.1056/NEJMoa1809697 30280658

[13] Nakanishi J, Wada Y, Matsumoto K, et al. Overexpression of B7-H1 (PD-L1) significantly associates with tumor grade and postoperative prognosis in human urothelial cancers. Cancer Immunol Immunother. 2007;56: 1173–1182. doi: 10.1007/s00262-006-0266-z 17186290 PMC11029839

[14] Nomi T, Sho M, Akahori T, et al. Clinical significance and therapeutic potential of the programmed death-1 ligand/programmed death-1 pathway in human pancreatic cancer. Clin Cancer Res. 2007;13: 2151–2157. doi: 10.1158/1078-0432.CCR-06-2746 17404099

[15] Zeng Z, Shi F, Zhou L, et al. Upregulation of circulating PD-L1/PD-1 is associated with poor post-cryoablation prognosis in patients with HBV-related hepatocellular carcinoma. PLoS One. 2011;6: e23621. doi: 10.1371/journal.pone.0023621 21912640 PMC3164659

[16] Hino R, Kabashima K, Kato Y, et al. Tumor cell expression of programmed cell death-1 ligand 1 is a prognostic factor for malignant melanoma. Cancer. 2010;116: 1757–1766. doi: 10.1002/cncr.24899 20143437

[17] Hamanishi J, Mandai M, Iwasaki M, et al. Programmed cell death 1 ligand 1 and tumor-infiltrating CD8+ T lymphocytes are prognostic factors of human ovarian cancer. Proc Natl Acad Sci U S A. 2007;104: 3360–3365. doi: 10.1073/pnas.0611533104 17360651 PMC1805580

[18] Pao W, Girard N. New driver mutations in non-small-cell lung cancer. Lancet Oncol. 2011;12: 175–180. doi: 10.1016/S1470-2045(10)70087-5 21277552

[19] Suda K, Onozato R, Yatabe Y, et al. *EGFR* T790M mutation: a double role in lung cancer cell survival? J Thorac Oncol. 2009;4: 1–4. doi: 10.1097/JTO.0b013e3181913c9f 19096299

[20] Ota K, Azuma K, Kawahara A, et al. Induction of PD-L1 expression by the EML4-ALK oncoprotein and downstream signaling pathways in non-small cell lung cancer. Clin Cancer Res. 2015;21: 4014–4021. doi: 10.1158/1078-0432.CCR-15-0016 26019170

[21] Gjerstorff ML. The Danish Cancer Registry. Scand J Public Health. 2011;39: 42–45. doi: 10.1177/1403494810393562 21775350

[22] Storm HH, Michelsen EV, Clemmensen IH, et al. The Danish Cancer Registry—history, content, quality and use. Dan Med Bull. 1997;44: 535–539. 9408738

[23] Jakobsen E, Rasmussen TR. The Danish Lung Cancer Registry. Clin Epidemiol. 2016;8: 537–541. doi: 10.2147/CLEP.S99458 27822096 PMC5094603

[24] Schmidt M, Schmidt SA, Sandegaard JL, et al. The Danish National Patient Registry: a review of content, data quality, and research potential. Clin Epidemiol. 2015;7: 449–490. doi: 10.2147/CLEP.S91125 26604824 PMC4655913

[25] Andersen TF, Madsen M, Jørgensen J, et al. The Danish National Hospital Register. A valuable source of data for modern health sciences. Dan Med Bull. 1999;46: 263–268. 10421985

[26] Erichsen R, Lash TL, Hamilton-Dutoit SJ, et al. Existing data sources for clinical epidemiology: the Danish National Pathology Registry and Data Bank. Clin Epidemiol. 2010;2: 51–56. doi: 10.2147/clep.s9908 20865103 PMC2943174

[27] Schmidt M, Pedersen L, Sørensen HT. The Danish Civil Registration System as a tool in epidemiology. Eur J Epidemiol. 2014;29: 541–549. doi: 10.1007/s10654-014-9930-3 24965263

[28] Hirsch FR, McElhinny A, Stanforth D, et al. PD-L1 immunohistochemistry assays for lung cancer: results from phase 1 of the Blueprint PD-L1 IHC Assay Comparison Project. J Thorac Oncol. 2017;12: 208–222. doi: 10.1016/j.jtho.2016.11.2228 27913228

[29] Rebelatto MC, Midha A, Mistry A, et al. Development of a programmed cell death ligand-1 immunohistochemical assay validated for analysis of non-small cell lung cancer and head and neck squamous cell carcinoma. Diagn Pathol. 2016;11: 95. doi: 10.1186/s13000-016-0545-8 27717372 PMC5055695

[30] ClinicalTrials.gov. Double Blind Placebo Controlled Controlled Study of Adjuvant MEDI4736 In Completely Resected NSCLC. ClinicalTrials.gov Identifier: NCT02273375. https://clinicaltrials.gov/ct2/show/NCT02273375. Accessed May 04, 2022.

[31] Chouaid C, Danson S, Andreas S, et al. Adjuvant treatment patterns and outcomes in patients with stage IB-IIIA non-small cell lung cancer in France, Germany, and the United Kingdom based on the LuCaBIS burden of illness study. Lung Cancer. 2018;124: 310–316. doi: 10.1016/j.lungcan.2018.07.042 30119925

[32] Osler M, Prescott E, Gottschau A, et al. Trends in smoking prevalence in Danish adults, 1964–1994. The influence of gender, age, and education. Scand J Soc Med. 1998;26: 293–298. doi: 10.1177/14034948980260041101 9868755

[33] Brody R, Zhang Y, Ballas M, et al. PD-L1 expression in advanced NSCLC: insights into risk stratification and treatment selection from a systematic literature review. Lung Cancer. 2017;112: 200–215. doi: 10.1016/j.lungcan.2017.08.005 29191596

[34] Yang CY, Lin MW, Chang YL, et al. Programmed cell death-ligand 1 expression in surgically resected stage I pulmonary adenocarcinoma and its correlation with driver mutations and clinical outcomes. Eur J Cancer. 2014;50: 1361–1369. doi: 10.1016/j.ejca.2014.01.018 24548766

[35] Zhang Y, Wang L, Li Y, et al. Protein expression of programmed death 1 ligand 1 and ligand 2 independently predict poor prognosis in surgically resected lung adenocarcinoma. Onco Targets Ther. 2014;7: 567–573. doi: 10.2147/OTT.S59959 24748806 PMC3990506

[36] D’Incecco A, Andreozzi M, Ludovini V, et al. PD-1 and PD-L1 expression in molecularly selected non-small-cell lung cancer patients. Br J Cancer. 2015;112: 95–102. doi: 10.1038/bjc.2014.555 25349974 PMC4453606

[37] Sun JM, Zhou W, Choi YL, et al. Prognostic significance of PD-L1 in patients with non-small cell lung cancer: a large cohort study of surgically resected cases. J Thorac Oncol. 2016;11: 1003–1011. doi: 10.1016/j.jtho.2016.04.007 27103510

[38] Sorensen SF, Zhou W, Dolled-Filhart M, et al. PD-L1 expression and survival among patients with advanced non-small cell lung cancer treated with chemotherapy. Transl Oncol. 2016;9: 64–69. doi: 10.1016/j.tranon.2016.01.003 26947883 PMC4800057

[39] Zhang J, Gao J, Li Y, et al. Circulating PD-L1 in NSCLC patients and the correlation between the level of PD-L1 expression and the clinical characteristics. Thorac Cancer. 2015;6: 534–538. doi: 10.1111/1759-7714.12247 26273411 PMC4511334

[40] Dietel M, Savelov N, Salanova R, et al. Real-world prevalence of PD-L1 expression in locally advanced or metastatic non-small cell lung cancer (NSCLC): the global, multicentre EXPRESS study. J Thorac Oncol. 2018;13(suppl): S74–S75. 10.1016/S1556-0864(18)30404-0. 31319978

[41] Aggarwal C, Rodriguez Abreu D, Felip E, et al. Prevalence of PD-L1 expression in patients with non-small cell lung cancer screened for enrollment in KEYNOTE-001, -010, and -024. Ann Oncol. 2016;27(suppl 6): 1060P. 10.1093/annonc/mdw378.14.

[42] Cooper WA, Tran T, Vilain RE, et al. PD-L1 expression is a favorable prognostic factor in early stage non-small cell carcinoma. Lung Cancer. 2015;89: 181–188. doi: 10.1016/j.lungcan.2015.05.007 26024796

[43] Tsao MS, Kerr KM, Kockx M, et al. PD-L1 immunohistochemistry comparability study in real-life clinical samples: results of Blueprint phase 2 project. J Thorac Oncol. 2018;13: 1302–1311. doi: 10.1016/j.jtho.2018.05.013 29800747 PMC8386299

[44] Rojas L, Cardona AF, Arrieta O, et al. PD expression in metastatic non-small cell lung cancer patients from Colombia (CLICAP). J Thorac Oncol. 2014;9(suppl 3): S199–S200.

[45] Pan W, Yang Y, Zhu H, et al. *KRAS* mutation is a weak, but valid predictor for poor prognosis and treatment outcomes in NSCLC: a meta-analysis of 41 studies. Oncotarget. 2016;7: 8373–8388. doi: 10.18632/oncotarget.7080 26840022 PMC4884999

[46] Shepherd FA, Domerg C, Hainaut P, et al. Pooled analysis of the prognostic and predictive effects of *KRAS* mutation status and *KRAS* mutation subtype in early-stage resected non-small-cell lung cancer in four trials of adjuvant chemotherapy. J Clin Oncol. 2013;31: 2173–2181. 10.1200/JCO.2012.48.1390. 23630215 PMC4881333

[47] Gooden MJ, de Bock GH, Leffers N, et al. The prognostic influence of tumour-infiltrating lymphocytes in cancer: a systematic review with meta-analysis. Br J Cancer. 2011;105: 93–103. doi: 10.1038/bjc.2011.189 21629244 PMC3137407

[48] Ishii H, Azuma K, Kawahara A, et al. Programmed cell death-ligand 1 expression and immunoscore in stage II and III non-small cell lung cancer patients receiving adjuvant chemotherapy. Oncotarget. 2017;8: 61618–61625. doi: 10.18632/oncotarget.18651 28977890 PMC5617450

[49] Epidemiology Frank L. When an entire country is a cohort. Science. 2000;287: 2398–2399. 10.1126/science.287.5462.2398. 10766613

