# Supplementary material for: Biomarker expression and survival in patients with non-small cell lung cancer receiving adjuvant chemotherapy in Denmark
Source: PLoS One. 2023 Apr 11;18(4):e0284037. doi: 10.1371/journal.pone.0284037 (PMC10089313; doi:10.1371/journal.pone.0284037)

## **S1 Fig.** **Network of population-based medical registries linkable on an individual level via the CPR number.** CPR, civil personal registration; NSCLC, non-small cell lung cancer.


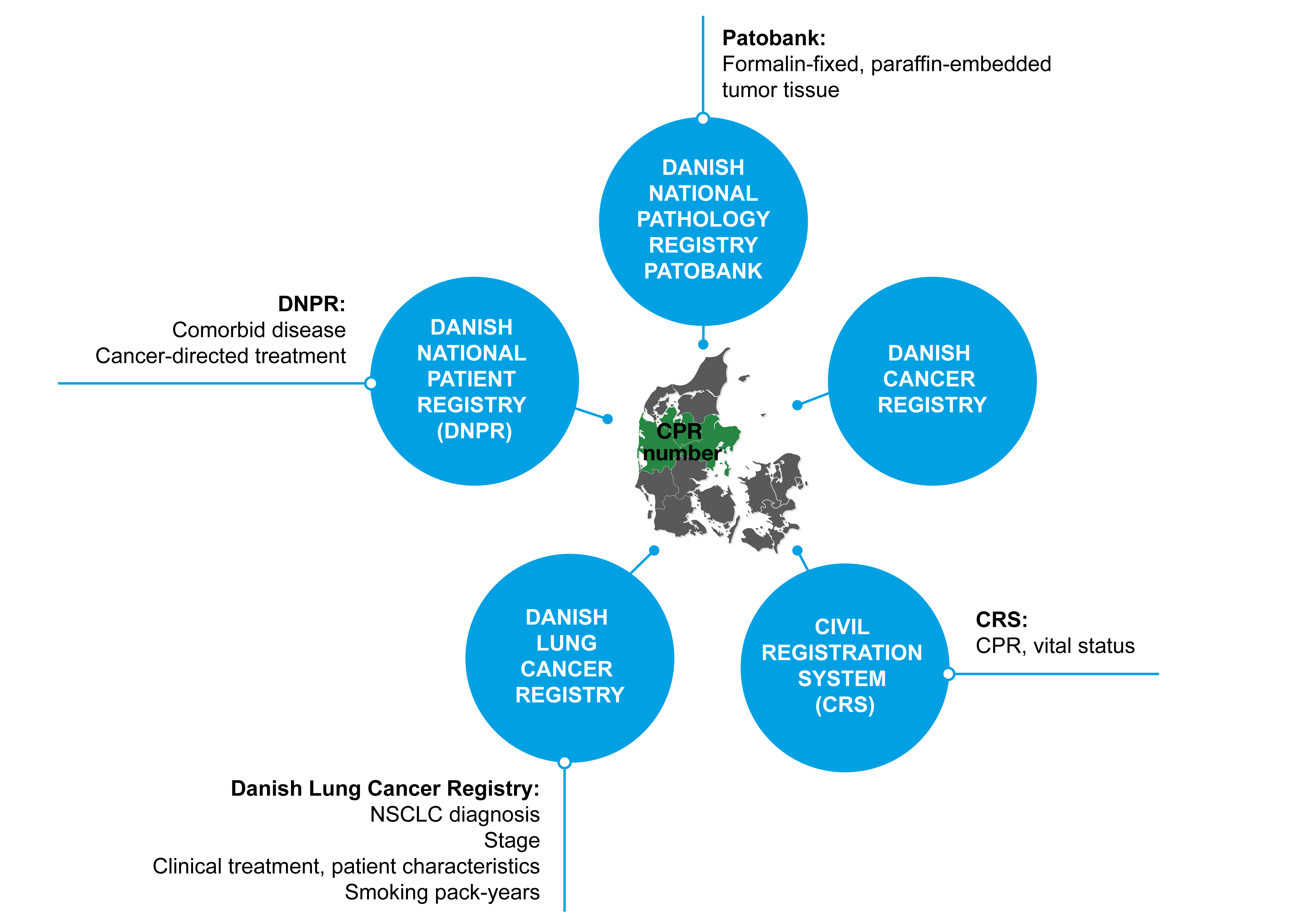

Supplement: S1 Fig — (DOCX) [file pone.0284037.s001.docx]
