# Supplementary material for: Biomarker expression and survival in patients with non-small cell lung cancer receiving adjuvant chemotherapy in Denmark
Source: PLoS One. 2023 Apr 11;18(4):e0284037. doi: 10.1371/journal.pone.0284037 (PMC10089313; doi:10.1371/journal.pone.0284037)
Supplement: S1 Table — (DOCX) [file pone.0284037.s002.docx]

## **S1 Table.** **Kaplan-Meier estimates of 25% survival (months, 95% CI) by biomarker status in patients with stage II or stage IIIA NSCLC receiving adjuvant chemotherapy.**

| Overall Survival | Stage II | Stage IIIA |
| --- | --- | --- |
| PD-L1 TC <25%  PD-L1 TC ≥25% | 31.7 (19.2–70.0)  27.3 (14.0–NR)^a^ | 17.2 (11.3–21.3)  21.9 (8.0–32.1) |
| PD-L1 IC <25%  PD-L1 IC ≥25% | 29.4 (16.2–66.2)  67.2 (14.0–NR)^a^ | 15.7 (11.6–22.2)  20.9 (7.3–26.8) |
| PD-L1 IC <1%  PD-L1 IC ≥1% | 31.7 (5.8–NR)^a^  30.5 (19.1–70.0) | 13.9 (3.4–19.0)  18.5 (14.1-24.0) |
| *EGFR* mutation  *EGFR* wild-type | 21.8 (6.3–NR)^a^  30.9 (19.1–67.2) | NR (15.7–NR)^b^  17.9 (12.7–21.7) |
| *KRAS* mutation  *KRAS* wild-type | 30.9 (13.4–66.0)  29.4 (19.1–85.1) | 18.5 (4.7–28.4)  15.7 (12.5–22.2) |

^a^The upper CI never reached the 25th percentile.

^b^Neither the survival estimate nor the upper CI reached the 25th percentile.
CI, confidence interval; *EGFR*, epidermal growth factor receptor; IC, immune cell; *KRAS*, V-Ki-Ras2 Kirsten rat sarcoma; NSCLC, non-small cell lung cancer; NR, not reached; PD-L1, programmed cell death ligand-1; TC, tumor cell.
