# Supplementary material for: Biomarker expression and survival in patients with non-small cell lung cancer receiving adjuvant chemotherapy in Denmark
Source: PLoS One. 2023 Apr 11;18(4):e0284037. doi: 10.1371/journal.pone.0284037 (PMC10089313; doi:10.1371/journal.pone.0284037)
Supplement: S2 Table — (DOCX) [file pone.0284037.s003.docx]

## **S2 Table.** **PD-L1 expression level by EGFR and KRAS mutation status in patients with stages II, and IIIA NSCLC receiving adjuvant chemotherapy.**

|  |  | | **Stage II** | | **Stage IIIA** | |
| --- | --- | --- | --- | --- | --- | --- |
|  |  |  | **PD-L1**  **TC <25%** | **PD-L1**  **TC ≥25%** | **PD-L1 TC <25%** | **PD-L1**  **TC ≥25%** |
| ***EGFR* status, n (%)** | | | | | | |
| Mutation |  |  | NR^a^ | NR^a^ | NR^a^ | NR^a^ |
| Wild-type |  |  | 115 (96.6) | 70 (94.6) | 92 (93.9) | 58 (98.3) |
| ***KRAS* status, n (%)** | | | | | | |
| Mutation |  |  | 28 (23.5) | 28 (37.8) | NR^b^ | NR^b^ |
| Wild-type |  |  | 88 (73.9) | 46 (62.2) | 76 (77.6) | 37 (62.7) |

^a^The numbers were too small to report.

^b^The numbers were too small to report for missing/invalid results, so results for the *KRAS*-mutated status could not be displayed. However, there were >5 patients in each stage with a *KRAS* mutation.

*EGFR*, epidermal growth factor receptor; *KRAS*, V-Ki-Ras2 Kirsten rat sarcoma; NSCLC, non-small cell lung cancer; NR, not reported; PD-L1, programmed cell death ligand-1; TC, tumor cell.
